# Ca^2+^ Dyshomeostasis Links Risk Factors to Neurodegeneration in Parkinson’s Disease

**DOI:** 10.3389/fncel.2022.867385

**Published:** 2022-04-14

**Authors:** Jianjun Xu, Etsuko Minobe, Masaki Kameyama

**Affiliations:** Department of Physiology, Graduate School of Medical and Dental Sciences, Kagoshima University, Kagoshima, Japan

**Keywords:** Parkinson’s disease, Ca^2+^ homeostasis, oxidative stress, ER stress, autophagy

## Abstract

Parkinson’s disease (PD), a common neurodegenerative disease characterized by motor dysfunction, results from the death of dopaminergic neurons in the substantia nigra pars compacta (SNc). Although the precise causes of PD are still unknown, several risk factors for PD have been determined, including aging, genetic mutations, environmental factors, and gender. Currently, the molecular mechanisms underlying risk factor-related neurodegeneration in PD remain elusive. Endoplasmic reticulum stress, excessive reactive oxygen species production, and impaired autophagy have been implicated in neuronal death in the SNc in PD. Considering that these pathological processes are tightly associated with intracellular Ca^2+^, it is reasonable to hypothesize that dysregulation of Ca^2+^ handling may mediate risk factors-related PD pathogenesis. We review the recent findings on how risk factors cause Ca^2+^ dyshomeostasis and how aberrant Ca^2+^ handling triggers dopaminergic neurodegeneration in the SNc in PD, thus putting forward the possibility that manipulation of specific Ca^2+^ handling proteins and subcellular Ca^2+^ homeostasis may lead to new promising strategies for PD treatment.

## Introduction

Parkinson’s disease (PD) is the second most common progressive neurodegenerative disorder in humans (Willis et al., 2010). It is characterized by motor dysfunction, cardinally manifested with resting tremor, slowness of movement, and muscle rigidity (Jankovic, 2008). Pathological analysis and clinical studies indicate that these motor PD symptoms result from the degeneration and loss of dopaminergic (DA) neurons in the substantia nigra pars compacta (SNc) (Kalia and Lang, 2016; Surmeier et al., 2017), a region in the midbrain involved in the regulation of movement and coordination of the human body. Unfortunately, since the mechanisms underlying PD pathogenesis remain elusive, there is currently no available cure for PD.

The precise causes of PD are largely unknown, and several etiologic factors, including aging, genetic and environmental factors, and gender (Ascherio and Schwarzschild, 2016), have been shown to increase the risk of PD development. One risk factor, or a combination of two or more factors, can distinctly contribute to PD pathogenesis (Pang et al., 2019; Vasta et al., 2021). Among these factors, aging is considered as the number one risk factor for PD development and progression (Hindle, 2010). Over 1% of the population worldwide suffers from PD at the age of 60, and this incidence rate increases to 5% in individuals over 85 years old (de Lau and Breteler, 2006), highlighting the impact of aging on the risk of PD. Genetic factors constitute another major risk factor for PD. Approximately 10–15% of all PD cases are caused by genetic mutations (Deng et al., 2018). Many mutations in genes associated with PD, including *GBA*, *LRRK2*, *PRKN*, *PINK1*, and *SNCA* (Kitada et al., 1998; Valente et al., 2001; Paisán-Ruíz et al., 2004; Lunati et al., 2018), have been identified. Moreover, several gene mutations have been shown to be the major cause of early onset PD (diagnosed in individuals younger than 45 years old) (Schrag and Schott, 2006), implying a superimposed effect of genetic factors on age-related PD pathogenesis. It is notable that PD is induced by a combination of aging with environmental and/or genetic factors (Pang et al., 2019; Vasta et al., 2021). Environmental factors include exposure to toxins and chemicals, which are associated with an increased risk of PD (Chen, 2018). Studies on the gender-based incidence of PD show that men are approximately 1.5∼2 times more likely than women to develop PD (de Lau et al., 2004), suggesting that gender is associated with PD incidence. The possible reasons for the higher risk of PD in men include lower neuroprotection by estrogen and higher chances of exposure to PD-related environmental factors. Moreover, other factors, such as head trauma and some medications have also been determined as the PD risk factors.

Although a number of PD risk factors have been identified, the molecular pathways of risk factor-related progressive neurodegeneration in PD require further elucidation. Dysfunction of the endoplasmic reticulum (ER), mitochondria, and lysosomes has been proposed to be the primary cause of neuronal death in PD patients (Michel et al., 2016; Sunanda et al., 2021). Dysfunction of the ER causes accumulation of unfolded proteins, which in turn triggers ER stress, resulting in an unfolded protein response (UPR). Impaired protein quality control and cellular Ca^2+^ dyshomeostasis have been implicated in the pathogenesis of PD (Xu et al., 2005; Lindholm et al., 2006; Kim et al., 2008). Mitochondrial dysfunction in pathological conditions leads to decreased ATP production, oxidative stress, and reduced calcium handling capacity, all of which are associated with neuronal damage in PD (Abou-Sleiman et al., 2006; Henchcliffe and Beal, 2008). Maintenance of a constant balance between synthesis and degradation of cellular constituents by the lysosomal system is essential for cellular homeostasis. Dysfunction of lysosomes results in the accumulation of aberrant proteins, lipids, and damaged organelles, especially mitochondria, leading to increased formation of toxic protein aggregates and excessive generation of reactive oxygen species (ROS), which are also crucially associated with neurodegeneration and neuronal death (Lin et al., 2019; Ray et al., 2021). Furthermore, recent studies have highlighted the essential role of the interactions between the ER, mitochondria, and lysosomes in the maintenance of normal cellular homeostasis (Raffaello et al., 2016; Rossini et al., 2021). However, dysfunction of one organelle has been shown to cause failure of the other two organelles, suggesting a switch-like mechanism or molecule in the cells. Once this mechanism or molecule is activated or reaches a threshold, virtuous interactions between organelles become vicious interplays, driving a vicious cycle between the ER, mitochondria, and lysosomes. Consistent with this hypothesis, dysfunction of these three important organelles has been shown to be a common feature in PD pathology (Perić et al., 2016; Ray et al., 2021). Ca^2+^, as the most important signaling molecule in neuronal cells, is crucial for the regulation of ER and mitochondrial and lysosomal functions and mediates the interactions between these organelles (Raffaello et al., 2016). Recently, dysregulation of intracellular Ca^2+^ handling, together with ER, mitochondrial, and lysosomal dysfunction, has been suggested as a common pathway in PD pathogenesis (Wojda et al., 2008; Michel et al., 2016; Zaichick et al., 2017). Therefore, it is worth considering whether Ca^2+^ dyshomeostasis links PD risk factors to neurodegeneration by triggering detrimental interactions between subcellular organelles.

We review recent findings and discuss how intracellular Ca^2+^ dyshomeostasis is induced by PD risk factors, which eventually leads to neurodegeneration and death of SNc DA neurons in PD.

## Ca^2+^ Handling in Neurons

**FIGURE 1:**
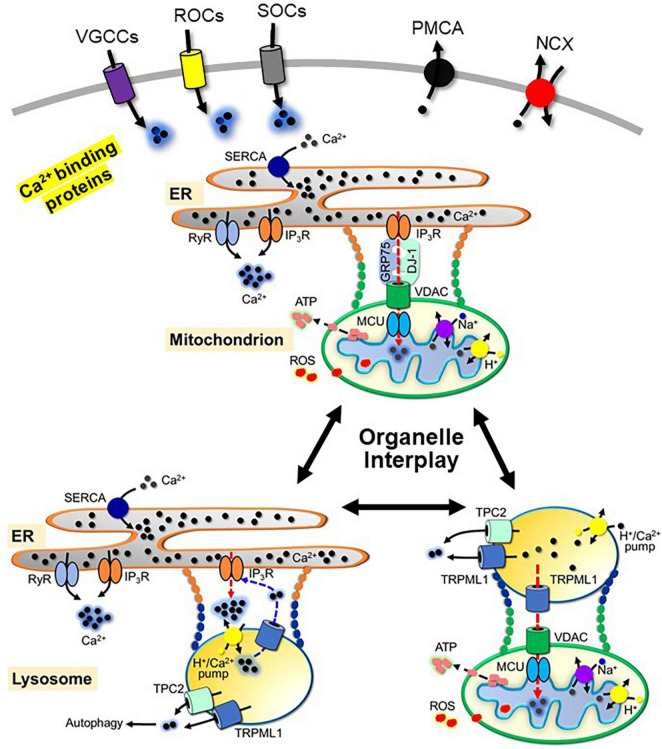
Schematic view of neuronal Ca^2+^ handling and the interplay between the ER, mitochondria and lysosomes. Ca^2+^ influx through voltage-gated Ca^2+^ channels (VGCCs), receptor-operated Ca^2+^ channels (ROCs) and store-operated Ca^2+^ entry channels (SOCs), and Ca^2+^ release from the ER through Ryanodine receptor (RyR) and inositol 1,4,5-triphosphate receptor (IP_3_R), from the mitochondria through Na^+^-Ca^2+^ exchanger and H^+^-Ca^2+^ exchanger, and from the lysosomes through the transient receptor potential channel mucolipin 1 (TRPML1) and two-pore channel 2 (TPC2) contribute to Ca^2+^ signaling. Mechanisms to regulate Ca^2+^ signaling include three pathways: extrusion via plasma membrane Ca^2+^-ATPase (PMCA) and Na^+^-Ca^2+^ exchanger (NCX); sequestration by Ca^2+^-binding proteins; uptake by the ER through sarco/endoplasmic reticulum Ca^2+^-ATPase (SERCA), uptake by the mitochondria through voltage-dependent anion channels (VDAC) and mitochondrial Ca^2+^ uniporter complex (MCU) and uptake by the lysosomes through lysosomal H^+^/Ca^2+^-exchanger. Organelle contacts between the ER, mitochondria and lysosomes include physical tethering and functional interactions. For simplicity, the components of each physical tether mentioned in the text are omitted. Ca^2+^ mediates functional interactions between organelles. Functional interactions between the ER and mitochondria are formed by the tetramer complex of VDAC, IP_3_R, deglycase 1 (DJ-1) and glucose-related protein 75 (GRP75). The interaction between the ER and lysosomes is mediated by IP_3_R and TRPML1. The interaction between the mitochondria and lysosomes is mediated by TRPML1, VDAC and MCU.

As the most ubiquitous and versatile second messenger in signaling pathways in neurons, Ca^2+^ is fundamental for neuronal development and survival by controlling diverse cellular processes including gene expression, synaptogenesis, synaptic transmission, energy production, membrane excitability, and neuronal plasticity (Kawamoto et al., 2012). Neuronal Ca^2+^ signals are induced after transient and reversible elevation of cytosolic Ca^2+^ concentration via Ca^2+^ influx through plasma membrane Ca^2+^-permeable channels and Ca^2+^ release from subcellular organelles (Brini et al., 2014) (Figure 1). The Ca^2+^-permeable channels in plasma membrane which mediate Ca^2+^ influx upon activation are categorized into three major groups (Brini et al., 2014): (1) voltage-gated Ca^2+^ channels (VGCCs). Neuronal VGCCs include L-type (Cav1.2-1.4), P/Q type (Cav2.1), N type (Cav2.2), R type (Cav2.3), and T type (Cav3.1-3.3) VGCCs. Cav1.2 and Cav1.3 are the main VGCC types in neurons. (2) Receptor-operated Ca^2+^ channels (ROCs). The main ROCs in neurons are glutamate and purinergic receptors. They are further divided into ionotropic and metabotropic receptors. Alpha-amino-3-hydroxy-5-methyl-4-isoxazole propionic acid-sensitive receptors (AMPARs) and N-methyl-d-aspartate sensitive receptors (NMDARs) are the principal types of ionotropic glutamate receptors. Ionotropic purinergic receptors (P2XRs) mediate Ca^2+^ influx and membrane depolarization, while metabotropic purinergic receptors (P2YRs) activate the G_*q/*11_ signaling pathway upon activation by ATP binding. (3) Store-operated Ca^2+^ entry channels (SOCs) activated by depletion of ER Ca^2+^ stores. SOCs communicate with the ER through interactions between STIM (the transmembrane stromal interaction molecules within the ER) and the ORAI subunit of SOC (Yuan et al., 2009).

In addition to Ca^2+^ influx, Ca^2+^ release from subcellular organelles contributes to Ca^2+^ signaling. ER and mitochondria are major intracellular Ca^2+^ stores in neurons, while lysosomes are also involved in the control of cellular Ca^2+^ homeostasis (Yang et al., 2019). Ca^2+^ release from the ER is mediated by the ryanodine receptor (RyR), which is activated by Ca^2+^ influx, and inositol 1,4,5-trisphosphate receptor (IP_3_R), which is activated by IP_3_ from G_*q/*11_ signaling pathways. Both RyR and IP_3_R are sensitive to cytosolic Ca^2+^ and oxidation states (Mak et al., 2001; Aracena-Parks et al., 2006; Xiong et al., 2006; Joseph et al., 2018). The mitochondrial system mainly consists of a Na^+^-Ca^2+^ exchanger (mNCX) and a H^+^–Ca^2+^ exchanger (Austin et al., 2017) responsible for Ca^2+^ release from the mitochondria. Ca^2+^ release from the lysosomes is mainly mediated through the transient receptor potential channel mucolipin 1 (TRPML1), which belongs to the mucolipin subgroup of the TRP ion channel family (Ramsey et al., 2006), and two-pore channel 2 (TPC2) (Calcraft et al., 2009).

Termination of cytosolic Ca^2+^ signals is mediated through three mechanisms: extracellular extrusion, uptake into intracellular organelles, and sequestration by Ca^2+^ binding proteins. Calcium efflux from the cytoplasm to the extracellular space is mainly mediated through the action of the plasma membrane Ca^2+^-ATPase (PMCA) and the Na^+^-Ca^2+^ exchanger (NCX) by using the energy stored in ATP and the chemical energy of the Na^+^ gradient, respectively. Uptake of Ca^2+^ into the ER is mediated by the sarco/endoplasmic reticulum Ca^2+^-ATPase (SERCA) in an ATP-dependent manner. The mechanism of Ca^2+^ uptake by the lysosomes is not completely understood. Melchionda et al. (2016) characterized the lysosomal H^+^/Ca^2+^-exchanger involved in lysosomal Ca^2+^ refilling in non-placental mammals. SERCA, PMCA, and ion exchangers are sensitive to the cellular oxidative status (Görlach et al., 2015). Increased ROS production inhibits the activity of these proteins. Mitochondria take up Ca^2+^ from the cytoplasm through voltage-dependent anion channels (VDAC) and mitochondrial Ca^2+^ uniporter complex (MCU), which is crucially involved in the sequestration of Ca^2+^ signaling (Shoshan-Barmatz and De, 2017). Ca^2+^ efflux and uptake into organelles by Ca^2+^ pumps and exchangers is a slow Ca^2+^ buffering process, while rapid Ca^2+^ buffering is mediated by intracellular calcium binding proteins (CaBPs). CaBPs are classified into Ca^2+^ sensors and Ca^2+^ buffer proteins (Burgoyne, 2007). Ca^2+^ sensor proteins, such as calmodulin, change their conformation upon Ca^2+^ binding and activate downstream signaling molecules. In contrast to Ca^2+^ sensor proteins, Ca^2+^ buffer proteins, such as calretinin, calbindin, or parvalbumin, quickly bind Ca^2+^ with different affinities when Ca^2+^ signals are generated in neurons and act as negative modulators of cytosolic calcium levels.

Ca^2+^ homeostasis is strictly controlled in healthy neurons. Disturbance in Ca^2+^ homeostasis leads to profound alterations in neuronal function. Especially, synapses, as the primary target of Ca^2+^, is highly vulnerable to Ca^2+^ dyshomeostasis. The axonal terminal of SNc DA neurons have high-energy requirement due to their extensive axonal arborization. Ca^2+^ dyshomeostasis in PD may initially attenuate nigrostriatal synaptic plasticity and DA release, and subsequently cause deconstruction of axons and loss of neuronal connectivity, preceding to the death of DA neurons and occurrence of gross PD motor symptom (Kouroupi et al., 2017; González-Rodríguez et al., 2021).

In contrast to other neurons, SNc DA neurons show two key features in cytosolic Ca^2+^ handling: (1) low Ca^2+^ buffering capacity. SNc DA neurons lack expression of the Ca^2+^ buffer protein calbindin (Dopeso-Reyes et al., 2014), which binds Ca^2+^ with rapid to intermediate kinetics. Calbindin is important for rapid restoration of basal Ca^2+^ levels within a cell soon after Ca^2+^ signal occurs. (2) SNc DA neurons are constitutively active in the absence of external input, generating slow, broad spikes that lead to a significant increase in resting cytosolic Ca^2+^ concentration. Although the mechanism underlying the pacemaking activity of SNc DA neurons is not completely clear, Cav1.3 channels have been shown to support pacemaking (Chan et al., 2007; Guzman et al., 2009). The combination of these two features in Ca^2+^ handling is shared by neurons at risk of neurodegeneration (Surmeier and Schumacker, 2013).

## Ca^2+^ Dyshomeostasis Mediates the Vicious Cycle Between Endoplasmic Reticulum Stress, Mitochondrial Oxidative Stress, and Impaired Autophagy in Parkinson’s Disease Pathogenesis

Ca^2+^ dyshomeostasis results from the disrupted balance between induction and termination of Ca^2+^ signaling, which is dynamically controlled by the Ca^2+^-handling molecules mentioned earlier under physiological conditions. Alterations in the activities or expression levels of Ca^2+^-handling proteins in pathological conditions causes dysregulation of Ca^2+^ handling, which has been extensively reported to contribute to neurodegenerative diseases (Mattson, 2004, 2007; Surmeier, 2007; Wojda et al., 2008; Marambaud et al., 2009). Considering the two features of Ca^2+^ handling (low Ca^2+^ buffering capacity and higher basal cytosolic Ca^2+^ level), SNc DA neurons are extremely susceptible to the increase in cytosolic Ca^2+^ induced by PD risk factors, such as aging, and genetic and environmental factors. Sustained Ca^2+^ elevation leads to neuronal damage through both direct and indirect pathways.

Excessive cytosolic Ca^2+^ can directly activate a variety of Ca^2+^-dependent enzymes, including phospholipases, proteases, endonucleases, and nitric oxide synthase (NOS). These activated enzymes contribute to neurodegeneration by damaging various cell structures, such as the membrane, cytoskeleton, and DNA, as well as through NO-induced oxidative stress (Tymianski and Tator, 1996).

In healthy neurons, cytosolic Ca^2+^ is tightly controlled within a physiological range by Ca^2+^-handling proteins, and Ca^2+^, in turn, regulates these Ca^2+^-handling proteins. In addition, Ca^2+^ plays a crucial role in the regulation of organelle functions, especially the ER, mitochondria, and lysosomes. Recently, increasing evidence has shed light on the central role of Ca^2+^ signaling in the crosstalk between the ER, mitochondria, and lysosomes (Raffaello et al., 2016). Prolonged Ca^2+^ dyshomeostasis under pathological conditions may feed a vicious interplay between ER, mitochondria, and lysosomes, which are hypothesized to participate in common pathways in neurodegeneration in PD.

Each subcellular organelle is physically separated from the cytoplasm, with its own unique role in cell function. Originally, subcellular organelles were considered as independent entities within a cell, however, recent discoveries have shown that there are dynamic interactions between organelles. Organelles are in close proximity, and they dynamically come in contact, with each other through membrane contact sites, which are enriched in signaling molecules and ion channels (Rowland and Voeltz, 2012; Prinz, 2014; Phillips and Voeltz, 2016; Muallem et al., 2017; Cohen et al., 2018; Lee et al., 2020; Rossini et al., 2021) (Figure 1). Membrane contact sites serve as “hotspots” for the transfer of signaling molecules and crosstalk between organelles, which are essential for cellular homeostasis. Disruption of the coordinated interactions between organelles results in the pathogenesis of various diseases, including PD.

### Mitochondrial-Endoplasmic Reticulum Contact

The most well-documented membrane contact site is the mitochondria-ER contact site (MERCS). In MERCS, the voltage-dependent anion channel (VDAC) in the outer mitochondrial membrane (OMM) is bridged with the ER membrane IP_3_R with deglycase 1 (DJ-1) and glucose-related protein 75 (GRP75). DJ-1 interacts with VDAC, IP_3_R, and GRP75. GRP75 links IP_3_R to VDAC. Together, IP_3_R, VDAC, GRP75, and DJ-1 form a functional tetramer complex (IP_3_R/GRP75/DJ-1/VDAC complex) at MERCS (Liu et al., 2019), which mediates Ca^2+^ transfer from the ER to the mitochondrial matrix via the inner membrane mitochondrial calcium uniporter (MCU) and likely ROS transfer from the mitochondria to the ER. MERCSs are regulated by multiple proteins in the ER and mitochondria membrane, which contribute to dynamic physical tethering between the organelles, and regulate the integrity, stability, size, and length of the MERCS (Csordás et al., 2018; Wilson and Metzakopian, 2021). Ca^2+^ dyshomeostasis may strengthen MERCS, by activation of Ca^2+^-dependent proteins in MERCS (Hajnóczky et al., 2014), which promotes physical and functional interaction between the ER and mitochondria. Enhanced MERCS has been reported to facilitate mitochondrial Ca^2+^ uptake, causing Ca^2+^ overload and mitochondrial dysfunction. Consistent with this hypothesis, there are increased ER–mitochondria contacts and upregulated MERCS-associated proteins, including IP_3_Rs, RyRs, and VDACs (Hedskog et al., 2013), and Ca^2+^ dyshomeostasis in neurodegenerative diseases (Wojda et al., 2008). Therefore, dynamic contacts between the ER and mitochondrial are beneficial for normal function of the ER and mitochondria. Deficits in MERCS lead to mitochondrial Ca^2+^ dyshomeostasis and dysfunction, and subsequent dopaminergic neuronal death in PD (Gómez-Suaga et al., 2018; Ray et al., 2021; Wilson and Metzakopian, 2021).

### Endoplasmic Reticulum-Lysosome Contact

The ER also dynamically contacts endolysosomal organelles. In the ER-lysosome contact sites, IP_3_Rs are highly clustered in the ER membrane (Atakpa et al., 2018). IP_3_R, together with lysosomal Ca^2+^-handling proteins such as the H^+^/Ca^2+^-exchanger, Ca^2+^ release channel TRPML1, and TPC2 form a functional unit in ER-lysosome contact sites, which contributes to ER-dependent lysosomal Ca^2+^ refilling. It has been reported that Ca^2+^ release from lysosomes via TRPML1 results in increased cytosolic Ca^2+^ concentration within the ER-lysosome contact sites, which subsequently activates IP_3_R to trigger massive Ca^2+^ release from the ER through the Ca^2+^-induced Ca^2+^ release (CICR) mechanism (Galione, 2015). Thus, ER and lysosomal Ca^2+^ release channels form an amplifying loop that controls lysosomal Ca^2+^ refilling. Both lysosomal Ca^2+^ refilling and Ca^2+^ release play essential roles in lysosomal functions, including late endosome-lysosome fusion (Pryor et al., 2000), lysosomal exocytosis, phagocytosis, membrane repair, and signal transduction (Reddy et al., 2001; Kinnear et al., 2004). The ER-lysosome contact is regulated by many proteins in the ER and endo-lysosomal membranes, which constitute dynamic physical interactions in the contact site (van der Kant and Neefjes, 2014). The ER-lysosome contact generates microdomain for lipid transport and Ca^2+^ transfer between the two organelles. Interestingly, the ER-endolysosome contact seems to be a Ca^2+^-sensitive structure. Epidermal growth factor stimulation triggers a spike in cytosolic Ca^2+^, and results in increased ER-endosome contacts formation, likely through the recruitment of some Ca^2+^-sensitive tethering proteins (Eden et al., 2010). Thus, the role of Ca^2+^ dyshomeostasis-induced increased ER-endolysosome contacts in PD pathogenesis warrants further investigation.

### Lysosome-Mitochondria Contact

Recently, several laboratories have observed physical dynamic contacts between lysosomes and mitochondria that are independent of mitophagy (Han et al., 2017; Valm et al., 2017; Wong et al., 2018). Lysosome-mitochondria contact has been shown to be regulated by the small GTPase Rab7 through the modulation of the contact site tethering and untethering dynamics (Wong et al., 2018). Rab7 exists in two forms in the cell: an active GTP-bound and an inactive GDP-bound form (Rab7-GTP and Rab7-GDP). Rab7-GTP at the lysosomal membrane promotes lysosome-mitochondria contact by interacting with unidentified effector proteins at the mitochondrial membrane (Wong et al., 2019). Rab7-GTP is inactivated by hydrolysis of its bound GTP into GDP upon activation of Rab7 GTPase, which causes lysosome-mitochondria untethering. Thus, lysosome-mitochondria contact depends on nucleotide binding to Rab7, which can be regulated by several cytoplasmic factors, such as guanine nucleotide exchange factors (GEFs) and GTPase activating proteins (GAPs). The contact site offers a platform for proteins at the mitochondrial and lysosomal membranes that regulates their interplay, such as metabolic exchange, and Ca^2+^ and ROS transfer between the two organelles. The lysosomal Ca^2+^ release channels TRPML1 and TPC2 together with mitochondrial VDAC in the outer membrane and MCU in the inner membrane form a functional unit that mediates Ca^2+^ transfer from the lysosome to the mitochondria (Peng et al., 2020). Dysregulation of mitochondria-lysosome contacts results in dysfunction of both the mitochondria and lysosomes and contributes to PD pathogenesis (Peng et al., 2020). Recent study shows prolonged lysosome-mitochondria contacts in neurons modeling PD, due to defect in GTP hydrolysis (Kim et al., 2021). Whether Ca^2+^ dyshomeostasis in PD is involved in facilitation of lysosome-mitochondria contacts remains to be elucidated.

### Ca^2+^ Dyshomeostasis and Mitochondrial Dysfunction

The main functions of mitochondria include ATP production, cytosolic Ca^2+^ buffering, and apoptosis control. Mitochondria synthesize ATP through the tricarboxylic acid (TCA) cycle and oxidative phosphorylation (Zhao et al., 2019). During oxidative phosphorylation, electrons are transported down the electron transport chain (ETC) and provide energy for the activity of the downstream respiratory H^+^ pump chain (respiratory complex I–IV). Respiratory complexes pump H^+^ out of the mitochondrial matrix and thus establish an H^+^ gradient and a mitochondrial membrane potential across the mitochondrial inner membrane. Mitochondrial membrane potential promotes ATP synthesis by complex V (Zorova et al., 2018) and supports MCU activity for Ca^2+^ uptake (Vais et al., 2020). Mitochondria are the major source of cellular ROS. ROS is produced in the mitochondria when electrons in the ETC are captured by oxygen, thus producing superoxide anion radicals. A deficit in ETC leads to excessive ROS production, which is known as mitochondrial oxidative stress (Bhatti et al., 2017). Ca^2+^ is a key regulator of mitochondrial function. The transient increase in cytosolic Ca^2+^ rapidly stimulates mitochondrial uptake, which helps to prevent Ca^2+^ overload in the cytosol and enhances ATP production to meet the increased metabolic demands by activation of Ca^2+^-dependent enzymes of both the TCA cycle and oxidative phosphorylation (Glancy and Balaban, 2012). However, a prolonged increase in cytosolic Ca^2+^ causes mitochondrial Ca^2+^ overload, resulting in the collapse of mitochondrial membrane potential, which reduces ATP production. Importantly, mitochondrial Ca^2+^ overload triggers mitochondrial oxidative stress by enhancing ROS production. Severe oxidative stress promotes the opening of the mitochondrial permeability transition pore (mPTP) and leads to the release of cytochrome *c*, triggering apoptosis (Peng and Jou, 2010). The mitochondria also release ROS to the cytoplasm via the mPTP.

### Ca^2+^ Dyshomeostasis and Endoplasmic Reticulum Stress

The ER is a major intracellular Ca^2+^ storage site in neurons involved in the generation and termination of cellular Ca^2+^ signaling. Three types of proteins are highly expressed in the ER with different functions in the control of cellular Ca^2+^ homeostasis: Ca^2+^ release channels (IP_3_R and RyR) contribute to cellular Ca^2+^ signaling generation by transporting Ca^2+^ from the ER lumen to the cytoplasm; ER resident Ca^2+^ binding proteins determine the ER Ca^2+^ buffering capacity; Ca^2+^ pumps (SERCA) contribute to sequestration of elevated cytosolic Ca^2+^ by pumping Ca^2+^ into the ER lumen. Both Ca^2+^ release channels and pumps are sensitive to cytosolic and ER luminal Ca^2+^ and ROS. Luminal Ca^2+^-binding proteins, such as calreticulin, calnexin, protein disulfide isomerases (PDI), and glucose-regulated protein 94 kDa (GRP94), regulate ER function not only by directly buffering luminal Ca^2+^, but also by regulating Ca^2+^ release channels, Ca^2+^ pumps, or ER proteins involved in protein synthesis and folding (Prins and Michalak, 2011).

The ER is also a “protein synthesis factory” responsible for the synthesis, folding, maturation, degradation, and translocation of secretory and transmembrane proteins (Braakman and Bulleid, 2011). Once translocated to the ER from ribosomes, the newly synthesized proteins undergo several post-translational modifications. Proper folding of proteins into their tertiary and quaternary structures is assisted by the coordinated work of chaperones, foldases, and cofactors (Yoshida et al., 2001). The optimal concentrations of ATP, Ca^2+^, and ROS regulate protein folding through disulfide bond formation (Hetz et al., 2011). Transportation of properly folded proteins inside vesicles to the surface of the cell membrane or extracellular space is strictly monitored by the ER protein quality control system (ERQC). If a protein is unfolded or misfolded, the protein is targeted for ER retention, which subsequently stimulates ER-associated degradation (ERAD) through proteolysis (Benyair et al., 2011). Depletion of ER Ca^2+^ stores, a decrease in ATP levels and an increase in the oxidative state lead to reduced protein-folding capacity of the ER, resulting in the accumulation and aggregation of unfolded or misfolded proteins, a condition termed ER stress (Bravo et al., 2013). ER stress triggers an adaptive response, called the unfolded protein response (UPR). UPR is a complex signaling pathway that controls adaptive processes through both transcriptional and non-transcriptional responses, regulating each aspect of the protein synthesis pathway, including protein folding, ER biogenesis, ERAD, protein entry to the ER, autophagy, and secretion, thus, UPR is a pro-survival response that is beneficial for immediate removal of unfolded or misfolded proteins and restoration of the normal function of the ER (Oslowski and Urano, 2011). However, prolonged ER stress leads to sustained UPR, converting a pro-survival response to pro-apoptotic signaling (Read and Schröder, 2021). Dyshomeostasis of cellular Ca^2+^ may induce depletion of ER Ca^2+^ stores by increasing Ca^2+^ release via RyR and IP_3_R, and cause Ca^2+^ overload in mitochondria, leading to reduced ATP production and oxidative stress, all of which contribute to ER stress and impaired UPR, thus initiating apoptosis.

### Ca^2+^ Dyshomeostasis and Lysosomal Dysfunction

Lysosomes are small organelles that function as the “recycling plant” of the cells, responsible for degradation of macromolecules (proteins, nucleic acids, lipids, and carbohydrates), damaged organelles and microorganisms (Trivedi et al., 2020). Lysosomes contain more than 100 membrane proteins that are involved in ion transport, autophagy, and membrane fusion and repair (Schwake et al., 2013). The acidic lumen of the lysosome is also abundant in hydrolases, including nucleases, proteases, lipases, glycosidases, sulfatases, and phospholipases (Luzio et al., 2014). These lysosomal hydrolases are responsible for breaking large molecules down to small molecules for recycling into the cytosol to form new macromolecules. They are also involved in the degradation of dysfunctional organelles, invaders, toxins, and pathogens that enter the cell via endocytic mechanisms. Autophagy is a cellular process through which cytosolic misfolded proteins or damaged organelles are directed to lysosomes for degradation. This process is important for the maintenance of normal cellular homeostasis by the clearance of dysfunctional proteins and organelles. Dysfunction of autophagy results in the accumulation and aggregation of aberrant proteins, such as α-synuclein, which is the major component of the Lewy bodies in PD neurons (Spillantini et al., 1997). Autophagy is highly regulated by Ca^2+^. Recent studies have shown that lysosomal Ca^2+^ release via TRPML1 is crucial for the regulation of autophagy. Ca^2+^ release through TRPML1 induces dephosphorylation and subsequent nuclear translocation of transcription factor EB (TFEB) by activation of Ca^2+^/calmodulin (CaM)-dependent phosphatase calcineurin. TFEB is a lysosomal master gene regulator that enters the nucleus and promotes expression of several genes associated with the regulation of the autophagic pathway (Medina et al., 2015). TRPML1 is a target of lysosomal membrane-resident mammalian target of rapamycin (mTOR). mTOR-mediated phosphorylation of TRPML1 inhibits TRPML1 activity, while mTOR function depends on Ca^2+^-CaM (Li et al., 2016). Thus, Ca^2+^ release through TRPML1 mediates a negative feedback regulation between mTOR and TRPML1. TRPML1 is also a ROS sensor whose activity is upregulated by ROS (Zhang et al., 2016). Once oxidative stress occurs, ROS induces Ca^2+^ release through TRPML1, which in turn enhances autophagy through the calcineurin-TFEB signaling pathway. Augmented autophagy promotes elimination of damaged mitochondria that produce excessive ROS and restores normal redox homeostasis. Mutations and dysfunction of TRPML1 have been implicated in the development of PD (Tsunemi et al., 2019). Dyshomeostasis of cytosolic Ca^2+^ may cause dysregulation of lysosomal Ca^2+^ homeostasis by triggering mitochondria-derived oxidative stress and ER stress. Excessive ROS stimulates lysosomal release via TRPML1, while ER stress-induced depletion of Ca^2+^ stores reduces lysosomal refilling, both of which may contribute to impaired lysosomal Ca^2+^ homeostasis, lysosomal dysfunction, and autophagy deregulation.

### Ca^2+^ Dyshomeostasis Converts Virtuous Interactions to Vicious Interplays Between the Endoplasmic Reticulum, Mitochondria, and Lysosomes

**FIGURE 2:**
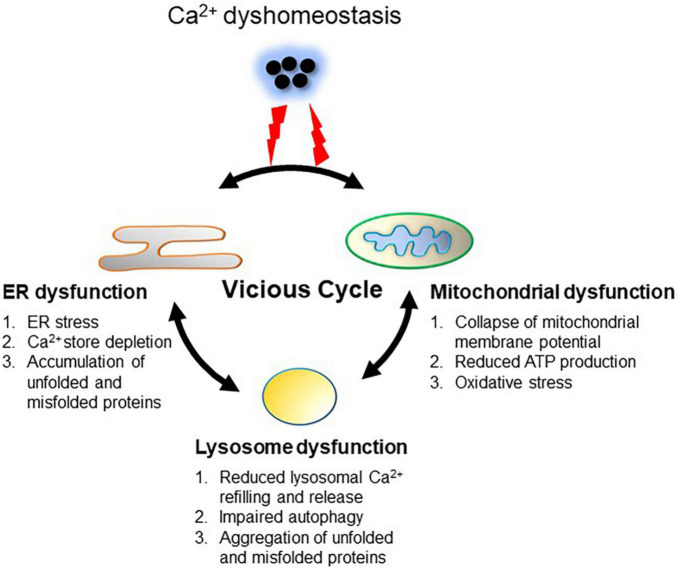
Ca^2+^ dyshomeostasis triggers a vicious cycle between the ER, mitochondria and lysosomes. Dysregulation of Ca^2+^ handling leads to intracellular Ca^2+^ overload. Prolonged excessive Ca^2+^ induces Ca^2+^ overload in the mitochondria through increasing mitochondrial uptake from the ER and cytoplasm, resulting in mitochondrial dysfunction (oxidative stress and reduced ATP production). ER dysfunction is induced by intracellular Ca^2+^ overload as well as increased ROS and reduced ATP from the mitochondria, resulting in ER stress, Ca^2+^ store depletion, and accumulation of unfolded or misfolded proteins. Dysfunction of the ER and mitochondria impairs lysosome functions through reduction of lysosomal Ca^2+^ refilling and subsequent Ca^2+^ release via TRPML1, which is tightly associated with impaired autophagy, leading to aggregation of abnormal proteins such as α-synuclein. Aggregation of α-synuclein in turn deteriorates ER and mitochondrial function. This vicious cycle between the ER, mitochondria and lysosomes perpetuates cellular Ca^2+^ dyshomeostasis.

Under physiological conditions, Ca^2+^ influx, via the plasma membrane Ca^2+^-permeable channel and subsequent Ca^2+^ release from subcellular organelles, induces Ca^2+^ signaling within a cell in response to various stimulations. Cellular Ca^2+^ signaling is a transient elevation of cytosolic Ca^2+^, which can be quickly terminated by extracellular extrusion, uptake into intracellular organelles, and sequestration by Ca^2+^-binding proteins. Transient elevation of cytosolic Ca^2+^ enhances the functions of the ER, mitochondria, and lysosomes. Elevated cytosolic Ca^2+^ moderately increases the Ca^2+^ load in the mitochondria, which promotes ATP production and ROS release from mitochondria (Glancy and Balaban, 2012). Ca^2+^ release from the ER and ROS release from the mitochondria in turn enhance lysosomal Ca^2+^ refilling and Ca^2+^ release via TRPML1, which augments autophagy signaling. All these processes are beneficial for cellular homeostasis. However, in pathological conditions, prolonged elevation of cytosolic Ca^2+^ converts the virtuous interplay between the ER, mitochondria, and lysosomes to a vicious cycle (Figure 2). Excessive cytosolic Ca^2+^ increases Ca^2+^ release from the ER and is transported to the mitochondria, causing mitochondrial Ca^2+^ overload (Santulli et al., 2015). Ca^2+^ overload leads to mitochondrial dysfunction, resulting in reduced ATP production and oxidative stress (Peng and Jou, 2010). Low ATP supply decreases ER Ca^2+^ uptake via SERCA, which requires ATP, while excessive ROS facilitates Ca^2+^ release from the ER through upregulation of RyR and IP_3_R, thus resulting in depletion of ER Ca^2+^ stores (Zhang et al., 2006). Reduced Ca^2+^ stores together with low ATP and higher levels of ROS cause ER dysfunction and impaired UPR, which enables accumulation of unfolded and misfolded proteins in the cells (Bahar et al., 2016). Depletion of ER Ca^2+^ stores attenuates lysosomal refilling and subsequent Ca^2+^ release via TRPML1, which impairs the autophagy signaling pathway, leading to accumulation of abnormal protein aggregates and damaged mitochondria within the cell (Shulman et al., 2011). Protein aggregates, such as α-synuclein aggregates, and accumulation of damaged mitochondria further enhance cellular oxidative stress (Reeve et al., 2015). Therefore, sustained cytosolic Ca^2+^ dyshomeostasis mediates the vicious interplay between ER stress, oxidative stress, and impaired autophagy. In contrast, dysfunction of the ER, mitochondria, and lysosomes further exacerbates Ca^2+^ dyshomeostasis. For example, depletion of ER Ca^2+^ stores activates SOCs, resulting in increased Ca^2+^ influx (Yuan et al., 2009). Toxic α-synuclein oligomers may integrate into the cell membrane to form a pore that allows Ca^2+^ influx (Kim et al., 2009). Excessive ROS has opposite effects on the activities of Ca^2+^-handling proteins responsible for the elevation and sequestration of cytosolic Ca^2+^. ROS facilitates plasma membrane Ca^2+^-permeable channels and organelle membrane Ca^2+^ release channels, however, it inhibits Ca^2+^-ATPase and Ca^2+^-transporters, which are responsible for pumping Ca^2+^ out of the cell or uptake by organelles, respectively (Görlach et al., 2015). Recently, accumulating evidence has shown that Ca^2+^ dyshomeostasis, ER stress, mitochondrial oxidative stress, and impaired autophagy typically appear in SNc DA neurons in PD (Abou-Sleiman et al., 2006; Calì et al., 2011; Michel et al., 2016; Sunanda et al., 2021), suggesting that dyshomeostasis is likely a common pathway in PD pathogenesis.

## Aging, Ca^2+^ Dyshomeostasis, and Parkinson’s Disease

Aging is an inevitable and irreversible biological process. The underlying mechanisms of aging remain unclear. Aging is a stochastic event with no defined triggering mechanisms and clear starting age, suggesting that advanced age itself may not be a problem, however, it increases the possibilities of exposure to environmental detrimental factors and gene mutations, which cause accumulation of cellular pathologies over time due to constant disruption of the balance between cellular damage and repair. Therefore body cells may become unable to maintain their homeostasis, in particular Ca^2+^ and redox homeostasis. Long-term exposure to toxic chemicals and ultraviolet light, chronic infections and inflammation, hormonal imbalance, psychological stress, and even bad lifestyle (dietary habits and sleep) may contribute to aging (Pyo et al., 2020).

Aging is now considered the greatest risk factor for PD. However, the mechanism linking aging to the loss of SNc DA neurons in PD is largely unknown. Ca^2+^ is crucial for the regulation of neuronal development, survival, and death, even slight alterations in cellular Ca^2+^ levels may have deleterious consequences on neurons. Recently, increasing evidence has shown that Ca^2+^ dyshomeostasis is involved in both aging and PD (Cortes et al., 2020), suggesting that dysregulation of Ca^2+^ handling is a common feature of aging and PD.

It has been widely reported that neuronal Ca^2+^ handling is dysregulated with age as a result of changes in the activity or cellular expression of proteins involved in the control of cellular Ca^2+^ homeostasis. Increased Ca^2+^ influx has been reported in aging neurons. AMPAR mediates Na^+^ influx in the presence of the GluR2 subunit in developing neurons, whereas AMPAR without GluR2 in aging neurons allows Ca^2+^ entry (Pandey et al., 2015). Upregulation of the expression level and activities of Cav1.3 channels in neurons has been reported in aging and PD (Veng and Browning, 2002; Hurley et al., 2013). α-synuclein aggregates in neurons, the hallmark of PD, are usually observed in aging too (Bobela et al., 2015). α-synuclein oligomers have been reported to form Ca^2+^-permeable channels, which promote an increase in cytosolic Ca^2+^ levels (Kim et al., 2009). Ca^2+^ release from the ER is increased, presumably due to the activation of IP_3_R and RyR by dysfunctional mitochondria-related ROS in aged neurons (Keller et al., 2004). In contrast, increased ROS and decreased ATP levels due to mitochondrial dysfunction in aging and PD inhibit SERCA, PMCA, and NCX, leading to impaired Ca^2+^ sequestration. Additionally, during brain aging, the expression levels of cellular Ca^2+^-binding proteins, such as calbindin and calretinin, are decreased in SNc DA neurons (Hurley et al., 2013), leading to lower Ca^2+^-buffering capacity. Therefore, with age, any detrimental factors or gene mutations that can directly impact neuronal Ca^2+^ handling or indirectly by affecting organelle functions may lead to Ca^2+^ dyshomeostasis and contribute to neurodegeneration and loss of neurons in PD.

## Gene Mutations, Ca^2+^ Dyshomeostasis, and Parkinson’s Disease

Since the first reports showing that *SNCA* was the primary causative gene involved in the early onset of familial PD (Polymeropoulos et al., 1997), an increasing number of genes have been reported as direct contributors or risk factors for PD. It is notable that the majority of PD cases are sporadic, with only 10-15% of cases having familial history (Deng et al., 2018). Therefore, there is now a general consensus that interactions between genetic factors and environmental exposure are major contributors to the etiology of PD, especially early onset PD (Cannon and Greenamyre, 2013). Moreover, accumulating clinical and pathological evidence shows that familial and sporadic forms of PD are often highly similar, suggesting that they may share common pathogenetic mechanisms (Pang et al., 2019). Indeed, similar to sporadic forms of PD, familial forms of PD are associated with genetic mutations that lead to mitochondrial dysfunction, oxidative stress, impaired autophagy, and accumulation of α-synuclein aggregates (Pang et al., 2019). As mentioned earlier, these pathological processes are regulated and triggered by Ca^2+^, which in turn exacerbates cellular Ca^2+^ dyshomeostasis, forming a vicious cycle in PD pathogenesis.

To date, there have been numerous genes whose mutations have been known to increase the risk of PD. Here, we focus on several reported genetic mutations that are well documented to be associated with PD pathogenesis and discuss how these mutations induce dysregulation of Ca^2+^ handling during PD progression.

### *SNCA* and *GBA*

*SNCA* is the first gene identified with mutations that contribute to early onset of familial PD. The *SCNA* gene encodes α-synuclein, a 140-amino acid protein. Most missense mutations, such as A30P, A53T, E46K, H50Q, and G51D are located at the amino terminus of α-synuclein and may cause familial PD (Klein and Westenberger, 2012). α-synuclein is natively unfolded and adopts an α-helical conformation when it binds to the cell membrane with its amino terminus (Burré et al., 2013), while the pathogenic missense *SNCA* variants tend to form stable β sheets that promote aggregation of α-synuclein, which is toxic to neurons (Bertoncini et al., 2005). Previous studies have shown that natively unfolded α-synuclein is selectively translocated into lysosomes for degradation (Cuervo et al., 2004). Decreasing lysosomal degradation or increasing the expression of unfolded and misfolded α-synuclein leads to aggregation.

The *GBA* gene encodes for a lysosomal glucocerebrosidase (GCase) that catalyzes the conversion of the sphingolipid glucosylceramide to ceramide and glucose. *GBA* mutations, such as E326K, are now considered as the single largest risk factor for idiopathic PD. *GBA* mutations cause decreased expression and activity of GCase, resulting in accumulation of glucosylceramide, which enhances α-synuclein aggregation by stabilizing soluble α-synuclein oligomers (Muñoz et al., 2021). Both *GBA* mutations and sporadic PD showed decreased GCase levels and increased α-synuclein aggregation.

Accumulation of α-synuclein aggregates has been reported in both familial and sporadic PD patients (Stefanis, 2012), suggesting a central role of this protein in PD pathogenesis. Aggregation of α-synuclein exacerbates cellular Ca^2+^ dyshomeostasis directly or indirectly, through the following mechanisms: (1) α-synuclein oligomers form Ca^2+^-permeable pores in the cell membrane that increase Ca^2+^ influx; (2) aggregated or misfolded α-synuclein interacts with and impairs mitochondrial complex I, causing mitochondrial dysfunction and oxidative stress in cells (Reeve et al., 2015), which indirectly causes Ca^2+^ dysregulation. When Ca^2+^ dyshomeostasis surpasses a certain threshold, it accelerates PD progression through perpetuating a vicious cycle between the mitochondria, ER, and lysosomes. Therefore, Ca^2+^ dyshomeostasis may link *SNCA* and *GBA* mutations to neurodegeneration and ultimately to the loss of neurons in PD.

### LRRK2

*LRRK2* encodes for leucine-rich repeat kinase 2 (LRRK2), a protein containing a GTPase and a kinase domain. *LRRK2* mutations are typically associated with late-onset familial PD (Tolosa et al., 2020). The G2019S variant has been reported to increase LRRK2 kinase activity (West et al., 2005). Although the exact mechanism underlying LRRK2-related PD pathogenesis is not fully clear, the G2019S variant has been shown to impair lysosomal functions through phosphorylation of its substrates, especially Rab proteins, which are required for all vesicular trafficking steps (MacLeod et al., 2006) as well as the interaction between mitochondria and lysosomes (Wong et al., 2018). Inactivation of Rab proteins by LRRK2-mediated phosphorylation is associated with lysosomal dysfunction in LRRK2-related PD. Interestingly, mutant LRRK2-mediated autophagic deficits can be reversed by inhibition of the endolysosomal Ca^2+^ release channel TPC2 (Hockey et al., 2015), suggesting that dysregulation of lysosomal Ca^2+^ handling is associated with LRRK2-related pathogenic processes.

### *PRKN* and *PINK1*

The *PRKN* (*PARK2*) gene encodes for the E3 ubiquitin ligase parkin, and mutations in parkin constitute the most common cause of early onset PD (Klein and Lohmann-Hedrich, 2007). Parkin contains a cysteine in its catalytic site that forms an intermediate thioester bond with ubiquitin, accelerating the covalent interaction between ubiquitin and the lysine residue of a substrate protein. Activation of parkin is mainly triggered by the binding of phosphorylated ubiquitin (pUbS65) or direct phosphorylation of parkin at serine 65 (pParkinS65). The mitochondria-resident protein, PTEN-induced putative kinase protein 1 (PINK1), mediates phosphorylation and activation of parkin; therefore, loss-of-function mutations in PINK1 lead to impaired parkin activity and are associated with a PD phenotype similar to that of mutated PRKN-related PD (Kamienieva et al., 2021). Parkin directs ubiquitin to its target substrates for proteasomal degradation. Parkin targets the mitochondria through interactions with PINK1. Once inactivated by PINK1, parkin ubiquitinates various mitochondrial regulatory proteins at the membrane surface for degradation, thus regulating mitochondrial functions, including mitochondrial fission and fusion, biogenesis, mitophagy, and ER-mitochondrial contacts. Mutations in *PRKN* lead to reduced parkin activity and subsequent mitochondrial dysfunction (Kamienieva et al., 2021).

## Environmental Factors, Ca^2+^ Dyshomeostasis, and Parkinson’s Disease

Although exposure to environmental factors (chemicals and toxins) has been associated with increased risk of PD, it is difficult to determine whether an environmental factor is the definitive cause of PD. In other words, each environmental factor may contribute to increased risk, but may not be sufficient to cause PD. It is widely accepted that PD results from complex interactions between two or more environmental factors, possibly independent of gene mutations (Ball et al., 2019). Curiously, few environmental factors directly affect Ca^2+^-handling proteins, suggesting that Ca^2+^ dyshomeostasis is indirectly triggered by cumulative cellular dyshomeostasis initiated by environmental factors, particularly mitochondrial dysfunction and oxidative stress. Once Ca^2+^ dysregulation reaches a threshold, it drives vicious interactions between the ER, mitochondria, and lysosomes, thus causing PD. In this review, some environmental factors that selectively target substantia nigra neurons are briefly discussed, including pesticides, heavy metals, and solvents.

### Pesticides

Pesticides are used in the agricultural industry to control pests and destroy weeds. Exposure to pesticides has been linked to increased risk of PD. Organochlorines and organophosphates are well-documented pesticides. One of the organochloride pesticides, dieldrin, preferentially targets SNc DA neurons and causes neurotoxic damage through the induction of oxidative stress and mitochondrial dysfunction (Kanthasamy et al., 2005). Application of the antioxidant N-acetyl cysteine protects from dieldrin-induced neurotoxicity by restoring normal ROS levels and mitochondrial membrane potential (Sharma et al., 2010). Rotenone is the main organophosphate component involved in increased PD risk through promotion of mitochondrial dysfunction by inhibiting mitochondrial complex I and ROS-activated aggregation of α-synuclein (Betarbet et al., 2000).

### Heavy Metals

There is a link between exposure to various metals and increased risk of PD. Occupational exposure to high-dose manganese during welding is associated with a form of parkinsonism, known as manganism, with symptoms such as tremors, gait abnormalities, and facial muscle spasms. Promotion of aggregation and cell-to-cell transmission of α-synuclein by manganese has recently been suggested to cause neurotoxicity in SNc DA neurons (Harischandra et al., 2019). Accumulation of either iron or copper in the midbrain has been linked to increased PD risk. Iron and copper, as redox metal ions, can elevate the oxidative state of DA neurons by contributing to the conversion of the superoxide anion and hydrogen peroxide into the hydroxyl radical through the Fenton and Haber-Weiss reactions (Collin, 2019). Long-term exposure to lead in the environment is also a risk factor for PD. Lead has been reported to decrease DA release by disrupting the dopaminergic system. In addition, lead is a pro-oxidant that can cause oxidative stress by enhancing ROS production, leading to cellular dyshomeostasis and subsequent neuronal damage (Neal and Guilarte, 2013).

### Organic Solvents

Exposure to organic solvents may also play an etiologic role in PD development. Organic solvents are commonly used in metal degreasing, dry cleaning, and as detergents. Trichloroethylene (TCE) is an industrial solvent, and exposure to TCE has been implicated as an environmental risk factor for PD. Although the exact mechanism underlying the effect of TCE on PD risk is not completely clear, TCE-associated mitochondrial dysfunction and deficits in complex I contribute to PD pathogenesis (Lock et al., 2013).

## Gender, Ca^2+^ Dyshomeostasis, and Parkinson’s Disease

Epidemiological studies have shown that PD is 1.5–2 times more frequent in men than in women (de Lau et al., 2004). The precise reasons for the gender difference in the incidence of PD are not fully understood. One hypothesis is that men are more likely to be exposed to PD-related environmental factors than women. However, meta-analysis reveals a similar incidence of PD in men and post-menopausal women, suggesting that female sex hormones may be involved in the protective effect in PD (Moisan et al., 2016). Increasing evidence suggests that estrogen may elicit a beneficial effect on SNc DA neurons in PD, although the underlying mechanism of estrogen actions remains to be clarified. Several aspects of estrogen action have been reported to help protect women from PD through genomic and non-genomic mechanisms. Estrogen enhances nigrostriatal DA synthesis and release (Jacobs and D’Esposito, 2011), which are tightly associated with Ca^2+^ signals. Whether the increased DA release is due to a direct action of estrogen or an indirect effect through an estrogen-induced increase in cytosolic Ca^2+^ requires further investigation. Recently, estrogenic control of mitochondrial function and biogenesis has drawn more attention. It has been extensively reported that estrogen enhances electron transport chain activity, maintains the mitochondrial membrane potential, increases ATP production, inhibits excessive ROS production, and prevents mitochondrial Ca^2+^ overload (Klinge, 2008, 2020; Lejri et al., 2018; Ventura-Clapier et al., 2019), which help maintain Ca^2+^ and cellular homeostasis. Furthermore, estrogen has been reported to support stable, helically folded α-synuclein tetramer that resists aggregation (Rajsombath et al., 2019), consistent with the findings that females show less accumulation of α-synuclein than males. However, it is notable that the beneficial effects of estrogen are related to healthy status of the neurons. Although estrogen protects healthy neurons against PD development, once neurodegeneration is induced, estrogen has little effect or even exacerbates disease (Morrison et al., 2006; Miller and Harman, 2017). Therefore, the precise mechanism of estrogen effect on SNc DA neurons remains to be further elucidated.

## Other Risk Factors, Ca^2+^ Dyshomeostasis, and Parkinson’s Disease

There are also other risk factors for PD, such as some medications which block the action of dopamine, and head trauma. In this review, the possible effect of head trauma on PD development is briefly discussed.

Growing evidence shows a clear link between head trauma and an increased risk of PD. Head injuries are common and can be caused by unexpected incidents, such as traffic accidents and falls, or some specific sport activities. The risk of PD due to head injuries is higher with more severe or recurrent injuries and loss of consciousness (Mckee and Daneshvar, 2015). Recent studies have shown that even mild traumatic brain injury (TBI) with loss of consciousness for more than 1 h may increase the risk of subsequent onset of PD by 1.5-fold, compared to controls (Gardner et al., 2018). The exact mechanism by which TBI increases the risk of PD is not clear. TBI-induced inflammation, metabolic dysregulation, and protein aggregation have been suggested as underlying mechanisms involved in the pathogenesis of PD (Delic et al., 2020). These studies showed that TBI accelerated α-synuclein aggregation in DA neurons (Acosta et al., 2015). Moreover, upregulation of LRRK2 has been found in the brains of mice following TBI, and inhibition of LRRK2 was neuroprotective in PD and TBI models (Bae et al., 2018). Rab is a substrate of LRRK2, which has been associated with PD progression; therefore, further studies are required to determine whether the contribution of TBI-induced LRRK2 upregulation to PD pathogenesis is mediated through Rab signaling.

## Summary

Parkinson’s disease is a chronic and progressive neurodegenerative disorder initiated by a number of risk factors, suggesting that risk factor-induced cellular dyshomeostasis accumulates over time. When cellular repair and compensatory systems cannot overcome sustained pathological injuries within cells, cell damage occurs. The ER, mitochondria, and lysosomes are crucial for the maintenance of cellular homeostasis, they interact with each other, and change their functions in response to signal stimulation. Ca^2+^ is the most important signaling molecule that participates in all cellular processes. Ca^2+^ transfer between subcellular organelles mediates coordinated and concerted interactions involved in cellular homeostasis. Under physiological conditions, cellular Ca^2+^ homeostasis is tightly controlled by Ca^2+^-handling proteins. However, in pathological conditions, prolonged impairment of cellular functions may directly or indirectly cause dysregulation of Ca^2+^ handling, resulting in cellular Ca^2+^ dyshomeostasis. SNc DA neurons are highly susceptible to an additional increase in cellular Ca^2+^, based on their features in Ca^2+^ handling. Sustained Ca^2+^ overload in SNc DA neurons may convert Ca^2+^ from a physiological regulatory factor to a pathological trigger. Indeed, growing evidence suggests that long-term excessive cytosolic Ca^2+^ contributes to ER, mitochondria, and lysosomal dysfunction in PD pathogenesis. Furthermore, recent studies support the hypothesis that Ca^2+^ dyshomeostasis is central in perpetuating the vicious cycle between the ER, mitochondria, and lysosomes, because increasing Ca^2+^ buffering capacity by overexpression of Ca^2+^-binding proteins or manipulation of subcellular Ca^2+^ handling can reduce oxidative stress, ameliorate autophagy, and decrease protein aggregation (Liu et al., 1997; Jung et al., 2019; Kang et al., 2021). Therefore, elimination of cellular Ca^2+^ overload and Ca^2+^ dyshomeostasis in subcellular organelles by application or expression of cell-specific and organelle-specific Ca^2+^-handling molecules may represent a promising strategy for PD treatment.

## Author Contributions

JX and MK wrote the manuscript. EM participated in discussion and collection of the manuscript. All authors approved the manuscript for publication.

## Conflict of Interest

The authors declare that the research was conducted in the absence of any commercial or financial relationships that could be construed as a potential conflict of interest.

## Publisher’s Note

All claims expressed in this article are solely those of the authors and do not necessarily represent those of their affiliated organizations, or those of the publisher, the editors and the reviewers. Any product that may be evaluated in this article, or claim that may be made by its manufacturer, is not guaranteed or endorsed by the publisher.
